# Genetic and environmental aetiology of the dimensions of Callous-Unemotional traits

**DOI:** 10.1017/S0033291715001919

**Published:** 2015-10-12

**Authors:** J. Henry, J.-B. Pingault, M. Boivin, F. Rijsdijk, E. Viding

**Affiliations:** 1 Laval University, Québec City, Canada; 2 University College London, London, United Kingdom; 3 Tomsk State University, Tomsk, Russian Federation; 4 King's College London, London, United Kingdom

**Keywords:** Callous-unemotional traits, conduct problems, genetic and environmental contributions, psychopathy

## Abstract

**Background:**

A Callous-Unemotional trait specifier (termed ‘Limited Prosocial Emotions’) was added to the diagnosis of conduct disorder in DSM-5. The Inventory of Callous-Unemotional Traits (ICU) is a comprehensive measure of these traits assessing three distinct, yet correlated dimensions – Callousness, Uncaring, and Unemotional – all thought to reflect the general Callous-Unemotional construct. The present study was the first to examine the degree to which the aetiology of these dimensions is shared *v*. independent.

**Method:**

Parent-reported ICU data from 5092 16-year-old twin pairs from the Twins Early Development Study were subjected to confirmatory factor analysis. Multivariate genetic modelling was applied to the best-fitting structure.

**Results:**

A general-specific structure, retaining a general factor and two uncorrelated specific factors (Callousness-Uncaring, Unemotional), provided the best fit to the data. The general factor was substantially heritable (h^2^ = 0.58, 95% CI 0.51–0.65). Unusually, shared environmental influences were also important in accounting for this general factor (c^2^ = 0.26, 95% CI 0.22–0.31), in addition to non-shared environmental influences. The Unemotional dimension appeared phenotypically and genetically distinct as shown by the substantial loadings of unemotional items on a separate dimension and a low genetic correlation between Unemotional and Callousness-Uncaring.

**Conclusions:**

A general factor, indicative of a shared phenotypic structure across the dimensions of the ICU was under substantial common genetic and more modest shared environment influences. Our findings also suggest that the relevance of the Unemotional dimension as part of a comprehensive assessment of CU traits should be investigated further.

## Introduction

Callous-Unemotional (CU) traits refer to a lack of guilt, disregard for others’ feelings and shallow display of emotions; characteristics that are the hallmark of psychopathy in adults (Cleckley, 1976; Hare, 2003) and also index youth at risk of developing psychopathy and persistent antisocial behaviour (Frick *et al.* 2014). CU traits characterize a subgroup of antisocial youth who show a particularly severe, aggressive, and stable pattern of conduct problems (Christian *et al.* 1997; Frick *et al.* 2003, 2005; Vincent *et al.* 2003; Frick & Dickens, 2006). This pattern holds for forensic, clinical and community samples (Frick *et al.* 2003; Kruh *et al.* 2005). Based on this extensive body of research, as well as findings of different aetiology and neurocognitive correlates of conduct problems in children with CU traits (Viding & McCrory, 2012), a CU specifier (termed Limited Prosocial Emotions) has been added to the diagnosis of conduct disorder in DSM-5 (APA, 2013).

The Inventory of Callous-Unemotional Traits (ICU; Frick, 2003) is a developmentally appropriate instrument commonly used to measure CU traits in children, adolescents and young adults. The ICU was originally designed to be a unidimensional instrument (Frick, 2003), but eleven empirical studies (using clinical and community samples) have since examined its factorial structure. Eight studies have utilized confirmatory factor analysis (CFA; Kimonis *et al.* 2008; Fanti *et al.* 2009; Roose *et al.* 2010; Byrd *et al.* 2013; Ezpeleta *et al.* 2013; Houghton *et al.* 2013; Ciucci *et al.* 2014; Waller *et al.* 2014; Hawes *et al.* 2014), two studies have employed both CFA and exploratory factor analysis (EFA; Essau *et al.* 2006; Feilhauer *et al.* 2012), and one study has used principal components analysis (PCA; Kimonis *et al.* 2013). These studies have tested various factorial structures, including a general-specific model (or bifactor model, as termed in past ICU studies), which has received the most consistent empirical support.

The general-specific factorial structure assumes that all items load on a general factor, and, in addition, load on specific factors. In this model, specific factors capture *residual variance* unaccounted for by the general factor. In other words, this general-specific structure posits that a general factor underlies individual differences in the ICU, but also that additional factors tapping unique dimensions are needed for a full representation of individual differences in ICU. These additional factors – by virtue of their *specific* quality – are orthogonal to each other and independent of a general factor. Previous studies have identified the following specific factors: (1) Callousness (lack of empathy for others and lack remorse for hurting others – e.g. ‘I do not care whom I hurt to get what I want’, ‘I do not feel remorseful when I do something wrong’); (2) Uncaring (lack of concern for others’ feelings or little desire to make others feel good – e.g. ‘I try not to hurt others’ feelings’, reverse scored, ‘I do things to make others feel good’, reverse scored) and; (3) Unemotional (impoverished, shallow and altered emotional experience and expression – e.g. ‘I hide my feelings from others’, ‘I am very expressive and emotional’, reverse scored; Essau *et al.* 2006; Kimonis *et al.* 2008; Fanti *et al.* 2009; Roose *et al.* 2010; Byrd *et al.* 2013; Ezpeleta *et al.* 2013; Ciucci *et al.* 2014; Waller *et al.* 2014). While this factorial structure has received substantial support in CFA studies, it is noteworthy that allocation of items to the various ICU factors has not been consistent across studies (Essau *et al.* 2006; Feilhauer *et al.* 2012; Houghton *et al.* 2013; Hawes *et al.* 2014).

Prior validation studies with youth and adult samples have shown that the ICU total score is internally consistent and shows expected associations with relevant external criteria, i.e. positive associations with conduct problems and delinquency, offence history, aggression, and psychosocial functioning, as well as negative associations with agreeableness, conscientiousness, openness, prosocial beliefs, empathy, and positive affect (Essau *et al.* 2006; Fanti *et al.* 2009; Kimonis *et al.* 2008; Roose *et al.* 2010). However, the internal consistencies of the dimensions vary considerably and typically range from poor to acceptable. Moreover, all three dimensions of the ICU have shown significant associations with other self-report measures of psychopathy, with associations involving Callousness and Uncaring dimensions being most robust (Kimonis *et al.* 2008; Roose *et al.* 2010).

Key distinctions have also emerged between the ICU dimensions. First, the Callousness and Uncaring dimensions are typically highly correlated, but the associations between these two dimensions and the Unemotional dimension are usually weaker (e.g. Roose *et al.* 2010). Second, both Callousness and Uncaring are associated with aggression in youth and Uncaring is also linked with the emotional deficits believed to be at the core of psychopathic traits (e.g. low psychophysiological responding; Essau *et al.* 2006; Fanti *et al.* 2009; Kimonis *et al.* 2008). In contrast to the Uncaring and Callousness dimensions, the Unemotional dimension has not demonstrated consistent or robust correlations with external correlates that are typically associated with psychopathy (Kimonis *et al.* 2008; Roose *et al.* 2010; Byrd *et al.* 2013). Contrary to Callousness and Uncaring, the Unemotional dimension is not related to delinquency and aggression (Kimonis *et al.* 2008; Byrd *et al.* 2013), and is also not consistently correlated with externalising behaviours and conduct disorder (Essau *et al.* 2006). These patterns of associations have led several authors (e.g. Kimonis *et al.* 2013) to consider two related possibilities: (1) the Unemotional items might index a dimension partly distinct from the Uncaring and Callousness dimensions; (2) the Unemotional dimension might not carry a *specific* risk for later psychopathy.

In brief, there are important phenotypic distinctions between the ICU dimensions. These distinctions could be due to differential genetic/environmental aetiology, but no study to date has examined the factorial structure of the ICU in a genetically informative design. To better understand the aetiology of the different trait dimensions underlying the broader CU construct, as well as their interrelations, we assessed a large, population-based sample of 16-year-old British twins using parent reports of the ICU. This study had two objectives: (1) to examine the phenotypic factorial structure of the ICU, and more importantly (2) to investigate the genetic/environmental aetiology of the ICU factors and their interrelation.

## Method

### Participants

The data in this study come from 5092 twin pairs from the Twins Early Development Study (TEDS) with 16-year parent-reported CU data (*N*_MZ_ = 1821; *N*_DZ_ = 3271). TEDS is a large population-based longitudinal study of twins born in England and Wales between 1994 and 1996. The sample and its history are described in detail elsewhere (Trouton *et al.* 2002; Oliver & Plomin, 2007; Haworth *et al*. 2013). Informed written consent was obtained from all of the families who agreed to take part in the study. The study and consent procedure were approved by the Institute of Psychiatry and Maudsley Ethics Committee. The average age of participants at the time of assessment was 16.32 years (s.d. = 0.68 years). Despite attrition, the TEDS sample that provided data at age 16 is closely matched to the UK population. Initially, 13 722 families returned data for first contact on TEDS. Of these families, 91.7% were of European descent, with 35.5% of mothers having A-levels or higher (A-levels are the national educational examination taken at 18 years in the UK, and refer to parental educational qualifications) and 43.1% of them being employed. Characteristics of the study sample were largely similar, with 95.9% of the families being of European descent, 42.7% of mothers having A-levels or higher and 49% of them being employed (see Supplementary Table S1 for details on sample demographics).

### Inventory of Callous-Unemotional Traits (ICU; Frick, 2003)

The ICU is a 24-item self-, parent- or teacher-report questionnaire designed to assess CU traits in youth. The content of the ICU was derived from the six-item CU scale of the Antisocial Process Screening Device (APSD; Frick & Hare, 2001), shown to designate a distinct subset of antisocial youth who display characteristics associated with the construct of psychopathy (Frick *et al.* 2000). The items are rated on a 4-point Likert scale ranging from 0 (*not at all true*) to 3 (*definitely true*). The parent-report version of the ICU was used in the current study. Internal consistencies ranged from acceptable (Callousness, *α* = 0.73; Unemotional, *α* = 0.72) to very good (Uncaring, *α* = 0.85; total scale, *α* = 0.88). Subscale-total correlations ranged between 0.67 and 0.88, and subscale correlations ranged between 0.34 and 0.62 (see Table 1 for descriptive statistics and correlations).

**Table 1.** Descriptive statistics and correlations on the Inventory of Callous-Unemotional Traits *(*ICU*)*, and demographic characteristics

				Correlations		
	Mean (s.d.)	Range	*α*	Total	CL	UC
Total	17.66 (9.28)	0–72	0.88			
CL	4.77 (3.64)	0–33	0.73	0.82**		
UC	7.70 (4.83)	0–24	0.85	0.90**	0.62**	
UE	5.18 (2.92)	0–15	0.72	0.67**	0.34**	0.42**

Total, Total ICU score; CL, Callousness subscore; UC, Uncaring subscore; UE, Unemotional subscore; *α*, Cronbach's alpha.

The ICU items were regressed on age and sex for all analyses. However, the raw ICU total scores and subscores were negatively, but weakly correlated with age (*r*_total_ = −0.05; *r*_callousness_ = −0.06; *r*_uncaring_ = −0.04; all *p*'s < 0.05), with the exception of the Unemotional subscale (*r*_unemotional_ = −0.03). Moreover, boys scored significantly higher on all scales with effect sizes (*d*) comprised between 0.28 and 0.42: total ICU scale (mean_boys_ = 19.87; mean_girls_ = 15.86; *d* = 0.33), on the Callousness (mean_boys_ = 5.30; mean_girls_ = 4.33; *d* = 0.28), Uncaring (mean_boys_ = 8.79; mean_girls_ = 6.82; *d* = 0.42) and Unemotional (mean_boys_ = 5.77; mean_girls_ = 4.71; *d* = 0.37).

*p < 0.05; **p < 0.01.

### Analyses

#### Confirmatory factor analyses

The factorial structure of the ICU was tested at the item level with CFA. Following previous work on the ICU (Essau *et al.* 2006; Kimonis *et al.* 2008, 2013; Fanti *et al.* 2009; Roose *et al.* 2010), we used a Robust maximum likelihood (MLR) estimator to account for item non-normality and the corresponding scaled statistics (Jöreskog & Sörbom, 1993). Following previous CFA research on the ICU (e.g. Essau *et al.* 2006; Kimonis *et al.* 2008), a set of alternative factorial structures was tested: (1) one factor; (2) three correlated factors; (3) two correlated factors; (4) hierarchical with three subfactors; (5) hierarchical with two subfactors; (6) general-specific with three specific factors; (7) general-specific with two specific factors. More details about the models are included in the Supplementary material. To account for non-independence of observations inherent to twin data, relevant specifications for twin dyads were included (e.g. variances and factor loadings equality-constrained across twins and zygosity; Olsen & Kenny, 2006). All items were age- and sex-regressed. Details about the fit indices are provided in the Supplementary material. On the basis of CFA analyses, we selected the best-fitting factorial structure for genetic modelling.

#### Genetic analyses

**Fig. 1.**
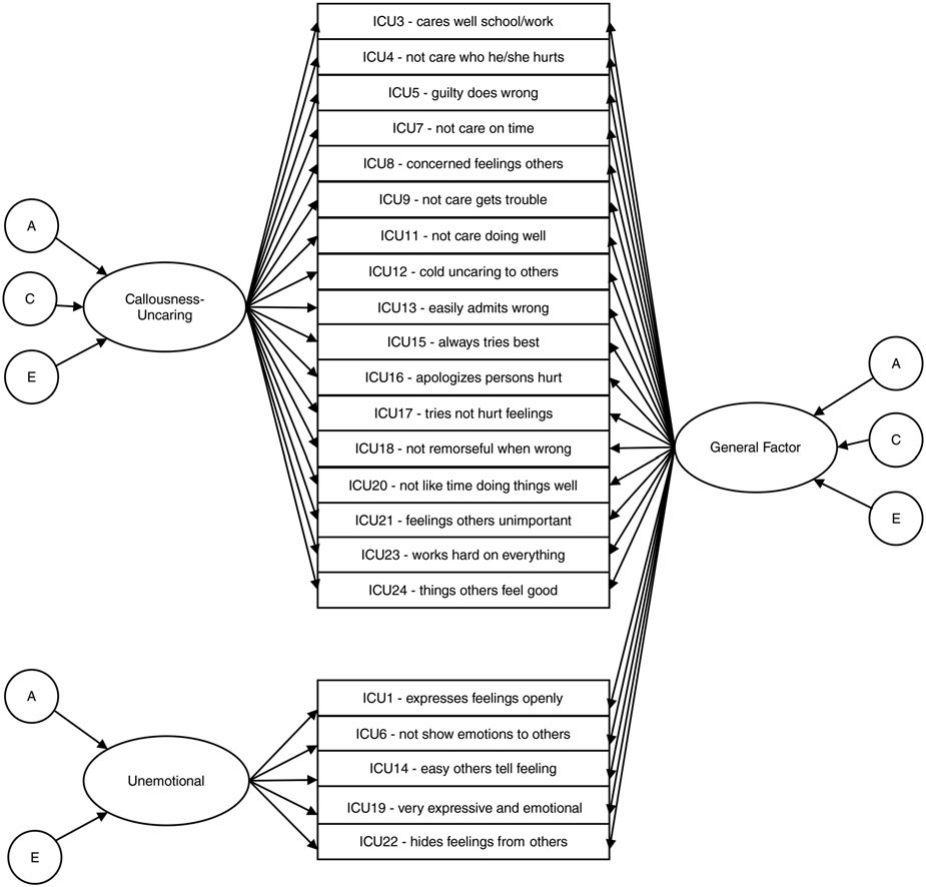
General-specific model of the Inventory of Callous–Unemotional traits (with multivariate genetic modelling on top of each factor). Residuals are not depicted for clarity.

Genetic modelling was applied to the best-fitting factorial structure of the ICU, i.e. the general-specific model including the Callousness-Uncaring and the Unemotional factors (see Fig. 1). The univariate ACE model decomposes the variance of a phenotype into additive genetic (h^2^), shared environmental (c^2^), and unique (or non-shared) environmental factors (e^2^; Neale & Cardon, 1992). In the present study, a multivariate genetic model was fitted to assess the genetic/environmental aetiology of all ICU factors. As seen in Fig. 1, this model may be described as a series of simultaneous univariate ACE models. This is because, in the general-specific model, no correlation exists between the general and the specific factors that could be decomposed in ACE components. The first part of the model assessed genetic/environmental contributions on the general factor. As all ICU items load on the general factor, this part of the model provides an indicator of aetiological overlap across the ICU. A second part of the model tested genetic/environmental contributions to each specific factor. As the specific factors represent residuals unaccounted for by the general factor and are orthogonal, this component specifies aetiological independence of each specific factor. More details about this model are included in the Supplementary material (see also a Cholesky decomposition of the ICU subscale scores in the Supplementary material; Supplementary Table S2). Missing data was handled using Full Information Maximum Likelihood (FIML; Arbuckle, 1996). All the analyses were conducted using the Structural Equation Modelling R package *Lavaan* (R 3.03; R Core Team, 2013; *Lavaan* 0.5-16; Rosseel, 2012).

## Results

Table 1 reports the descriptive statistics, internal consistencies, ranges and correlations of the ICU scores.

### Confirmatory factor analyses

Fit indices for the CFA analyses are presented in Table 2. The three-factor general-specific model showed a better fit than other models (i.e. one factor, hierarchical with three subfactors, hierarchical with two subfactors, three correlated factors, two correlated factors). However, the best model fit was attained with the two-factor general-specific model, which indicates that item allocation to two factors – rather than three – provides a better fit to the ICU data.

**Table 2.** Fit indices comparing alternative confirmatory factor models for the Inventory of Callous-Unemotional Traits

	Fit indices (scaled where applicable)				
Model	AIC	χ^2^ (df)	CFI	RMSEA	SRMR
Hierarchical (three factors)	372 612	20 806 (1953)*	0.779	0.064	0.075
Hierarchical (two factors)	372 610	20 816 (1954)*	0.779	0.064	0.079
One factor	372 606	20 838 (1956)*	0.779	0.064	0.075
Three factors	364 533	15 372 (1928)*	0.843	0.054	0.068
Two factors	356 167	15 781 (1946)*	0.838	0.055	0.074
Three-factor general-specific	356 206	9723 (1928)*	0.909	0.041	0.066
Two-factor general-specific	353 202	7635 (1930)*	0.933	0.035	0.062

AIC, Akaike's Information Criterion; CFI, Comparative fit index; df, degrees of freedom; RMSEA, root mean square error of approximation; SRMR, standardized root mean square residual.

For each model, all items were regressed on child gender and age. Each model was analysed without items 2 and 10.

* *p* *<* 0.001.

We further examined the data to determine whether particular items contributed to suboptimal model fit. Item-total correlations indicated that items 2 (*r* = 0.16; ‘What he/she thinks is right and wrong is different from what other people think’) and 10 (*r* = −0.15; ‘He/she does not let his/her feelings control him/her’) from the Callousness-Uncaring subset were essentially unrelated to the remaining items on the scale. Previous studies reported low item-total correlations and/or factor loadings for these two items (e.g. Essau *et al.* 2006; Hawes *et al.* 2014). We suspect that item 2 is unspecific as CU traits are only one among many reasons why the notion of right and wrong may differ in an individual compared to others. Moreover, the wording of item 10 could be interpreted as indicating emotional repression or a capacity for self-control. As in Kimonis *et al.* (2008) and Hawes *et al.* (2014), we withdrew these items from our analyses. Each model presented in Table 2 was analysed *without* these items.

Table 3 presents the factor loadings for the general-specific model with two specific factors. On average, loadings of the Callousness-Uncaring items were weaker on the specific factor (−0.05 to 0.50) than on the general factor (0.21–0.61), which indicates that these items were well represented by the general factor. All items which loaded highly on the specific Callousness-Uncaring factor also loaded highly on the general factor. These items were very similar in content in that they provided indicators of the extent to which the target child tries to do his/her best at school or at work [e.g. item 3: ‘He/she cares about how well he/she does at school or work. (R)’; item 20: ‘He/she does not like to put the time into doing things well.’]. The Unemotional items loaded highly and uniformly on their specific factor.

**Table 3.** Factor loadings for the general-specific model of the Inventory of Callous–Unemotional Traits *(*ICU*)*

Item	Total	CL-UC	UE
**Callousness-uncaring**			
*Callousness*			
2. What he/she thinks is wrong is different from what other people think*	–	–	
4. He/she does not care who he/she hurts to get what he/she wants	0.22	−0.02	
7. He/she does not care about being on time.	0.22	0.15	
8. He/she is concerned about the feelings of others (R)	0.58	−0.04	
9. He/she does not care if he/she gets into trouble (R)	0.52	0.09	
10. He/she does not let his/her feelings control him/her*	–	–	
11. He/she does not care about doing things well	0.21	0.25	
12. He/she seems very cold and uncaring to others	0.20	−0.04	
18. He/she does not feel remorseful when he/she does something wrong	0.32	−0.02	
20. He/she does not like to put the time into doing things well	0.28	0.39	
21. The feelings of others are unimportant to him/her	0.26	−0.03	
*Uncaring*			
3. He/she cares about how well he/she does at school or work (R)	0.38	0.45	
5. He/she feels bad or guilty when he/she does something wrong (R)	0.52	0.02	
13. He/she easily admits to being wrong (R)	0.49	−0.04	
15. He/she always tries his/her best (R)	0.43	0.45	
16. He/she apologizes to persons he/she hurt (R)	0.61	−0.05	
17. He/she tries not to hurt others feelings (R)	0.43	−0.05	
23. He/she works hard on everything he/she does (R)	0.43	0.50	
24. He/she does things to make others feel good (R)	0.53	0.01	
**Unemotional**			
1. He/she expresses his/her feelings openly (R)	0.34		0.53
6. He/she does not show his/her emotions to others	0.20		0.28
14. It is easy for others to tell how he/she is feeling (R)	0.36		0.44
19. He/she is very expressive and emotional (R)	0.20		0.43
22. He/she hides his/her feelings from others	0.21		0.41

CL, Callousness factor; UC, Uncaring factor; UE, Unemotional factor; (R), reverse-scored item.

Factors are highlighted in bold. Subscales are in italic.

* These items were withdrawn from analyses.

### Genetic analyses

Results from the genetic analyses conducted on the ICU factors are presented in Table 4, which includes estimates of genetic (h^2^), shared environmental (c^2^) and unique environmental contributions (e^2^) to each factor. Intraclass correlations (ICCs) for the total ICU score (ICC_MZ_ = 0.82, 95% CI 0.80–0.83; ICC_DZ_ = 0.47, 95% CI 0.44–0.50) and for the subscale scores (Callousness-Uncaring: ICC_MZ_ = 0.83, 95% CI 0.81–0.84; ICC_DZ_ = 0.49, 95% CI 0.46–0.52, Unemotional: ICC_MZ_ = 0.70, 95% CI 0.67–0.72; ICC_DZ_ = 0.29, 95% CI 0.26–0.32) were indicative of moderate to high heritability and this was confirmed by genetic modelling (general factor: h^2^ = 0.58; Callousness-Uncaring: h^2^ = 0.70; Unemotional: h^2^ = 0.79). The shared environment component was only significant for the general factor (c^2^ = 0.26). Modest to moderate contributions of unique environment were also found (general factor: e^2^ = 0.16; Callousness-Uncaring: *e*^2^ = 0.30; Unemotional: e^2^ = 0.21). Thus, the present results show a genetic contribution to the general factor, which suggests shared genetic variance across the whole set of ICU items. Over and above the effects to the general factor, unique genetic contributions were found on the Callousness-Uncaring and Unemotional factors.

**Table 4.** Genetic modelling on the CU general, Callousness-Uncaring, and Unemotional factors derived from the two-dimension general-specific model for the Inventory of Callous–Unemotional Traits (ICU)

	Parameter estimates		
	h^2^ (95% CI)	c^2^ (95% CI)	e^2^ (95% CI)
CU general	**0.58** (**0.47–0.66)**	**0.26** (**0.20 to 0.35)**	**0.16** (**0.13–0.19)**
CL-UC	**0.70 (0.62–0.76)**	0.00 (−0.11 to 0.10)	**0.30 (0.26–0.35)**
UE	**0.79 (0.73–0.83)**	–	**0.21 (0.17–0.27)**

CU general, General Callous–Unemotional factor; CL-UC, Callousness-Uncaring factor; UE, Unemotional factor; CI, Confidence interval; h^2^, additive genetic factors; c^2^, shared environmental factors; e^2^, non-shared (or unique) environmental factors or measurement error.

CL–UC, and UE factors represent residuals unaccounted for by the CU general factor. An ACE structure was tested on the CU general and CL–UC factors, while an AE structure was tested on the UE factor. Sibling interaction effects are included for the Unemotional factor.

Statistically significant parameters are highlighted in bold.

Finally, results from our Cholesky decomposition conducted on the ICU subscale scores (see the Supplementary material) indicate that 78% of the genetic variance of the Unemotional score was specific to this factor and not explained by genetic factors underlying the Callousness-Uncaring subscore. This analysis further underscores that the Unemotional items define a dimension partly separate from dimensions that are more representative of the core construct of interest (i.e. those tapped by items from the Callousness and Uncaring subscales).

## Discussion

In a large youth sample, the present study investigated the phenotypic structure and the genetic/environmental aetiology of Callous-Unemotional traits, as assessed by the ICU. A general-specific model – comprising a general factor and two specific factors (Callousness-Uncaring, Unemotional) – fitted the data best. All items, particularly those from the Callous-Uncaring dimension, loaded on the general factor. Twin analyses, conducted for the first time with this instrument, indicated that the general factor was substantially heritable. This suggests a substantial degree of common genetic contributions across the CU construct as measured by the ICU. Noticeably, shared environmental influences also contributed to the general factor. In addition, our analyses indicated that in contrast to the other dimensions, the Unemotional dimension was partly distinct both phenotypically and also in terms of its aetiology, in particular genetic influences. We will first discuss the general phenotypic and genetic/environmental structure of the ICU before moving to the distinctions between the specific factors, Callousness-Uncaring and Unemotional.

Previous studies have reported that a general-specific model with three specific factors fitted best the ICU in adolescent (Essau *et al.* 2006; Kimonis *et al.* 2008; Fanti *et al.* 2009; Roose *et al.* 2010) and young adult samples (Byrd *et al.* 2013; Kimonis *et al.* 2013) – both in community (Essau *et al.* 2006; Fanti *et al.* 2009; Roose *et al.* 2010; Byrd *et al.* 2013; Kimonis *et al.* 2013) and clinical youth (Kimonis *et al.* 2008). Our findings also suggest that a general-specific model fits the data best, but with two (Callousness-Uncaring and Unemotional), rather than three factors.

Past behavioural genetic research on CU traits has reported consistent results of moderate to strong heritability of CU traits (see Viding & McCrory, 2012). Accordingly, our analyses showed that the general factor is substantially heritable. As all ICU items load on this general factor, this implies that there is a degree of shared genetic risk across the whole set of ICU items. Notably, common genetic effects on the whole ICU scale may imply contributions of one specific class of risk genes, possibly linked to a limited number of intermediate phenotypes (e.g. reactivity of brain networks critical for affective/empathic processing) in the development of CU traits. This one-faceted genetic aetiology may also imply a limited number of temperamental and cognitive-affective precursors (and early treatment targets) to CU traits. Yet, the general-specific model also indicates that the ICU measures specific features that differ in terms of their psychometric features and links to a general construct. In line with this, unique genetic contributions were also found for the Callousness-Uncaring and Unemotional factors.

Our twin analyses also revealed that shared environmental influences, i.e. influences that make twins more similar, contributed to the general factor but not to the specific factors. The magnitude of the influence on the general factor (26%) is noticeable as a meta-analysis found estimates ranging from 12% to 21% for different types of mother-reported child and adolescent psychopathology symptoms (Burt, 2009). While a heritable component was expected for CU traits, shared environmental variance is not commonly found in twin research on CU traits (see Viding & McCrory, 2012). There may be several reasons for this finding. First, parent reports more commonly detect shared environmental effects (Burt, 2009). Second, the ICU measure itself comprises many items and may be thus more accurate and sensitive in its assessment of CU traits (and their aetiology) than shorter CU measures deployed in previous studies. These shorter measures may have been more prone to measurement error, which would end up in the non-shared environmental variance component). Third, the use of a general-specific structure may also have reduced measurement error on the general factor, enabling a better detection of the shared environmental component. If genuine, these shared environmental estimates may reflect identifiable systematic and relatively persistent influences on psychopathology during childhood (Burt, 2009). As such, our results raise the prospect of identifying reliable shared environmental influences on carefully measured CU traits. Chaos in the home (i.e. disorganized household) is an example of one such possible shared environmental influence.

Non-shared environment (i.e. child specific experiences that make the twins different and/or measurement error) also accounted for variance on all ICU factors, in particular for the specific factors. Association with a deviant peer group in adolescence is a strong candidate risk factor that may account for some of these non-shared environment influences on CU traits (Kimonis *et al*. 2004), especially as CU traits were assessed at age 16 years in the present study.

In the present study, we examined the previously untested possibility that two rather than three specific factors were sufficient to account for the ICU structure. A structure with two specific factors – one grouping Callousness and Uncaring items together and the other including Unemotional items – provided a better fit than a structure with three specific factors. Although previous studies had not tested this factorial structure, there were several indicators of its relevance, notably stronger associations between Callousness and Uncaring dimensions with each other than with the Unemotional dimension, and uncertainty as to where – either to the Callousness or the Uncaring subscale – some items should be allocated. Hawes *et al.* (2014) proposed that the separate dimensions of Uncaring and Callousness found in past studies may have been an artefact of item wording, with the former having items largely worded in the negative direction and the latter including items largely worded in the positive direction. A recent study (Ray *et al.* 2015) provided empirical support for this idea, showing that positively worded items, composing most of the Callousness subscale: (1) are less endorsed than negatively worded items; (2) discriminate best at higher levels of CU traits in item response theory (IRT) analyses, and; (3) are more closely related to antisocial/aggressive behaviour (Ray *et al.* 2015). It was therefore concluded that the general-specific structure identifying three specific factors may be an artefact of different item properties (Ray *et al.* 2015). The present results, indicating that regrouping Callousness and Uncaring items provides a more parsimonious solution, are clearly in line with this conclusion. Several elements distinguish the two specific factors identified by our factor analysis. First, the general factor seems to account well for the Callousness-Uncaring items, as suggested by the presence of strong loadings onto the general factor. The few items which *also* loaded on the specific Callousness or Uncaring factors seemed to reflect behaviours that are related to lack of conscientiousness (e.g. ‘He/she does not care about doing things well’, ‘He/she works hard on everything he/she does’). This may not be surprising, considering psychopathy is closely related to low conscientiousness in young adults (Paulhus & Williams, 2002). Whether this specific factor taps into a construct closely related to conscientiousness should be further tested in future studies that include a measure of conscientiousness.

Second, while the general factor accounted well for the Callousness-Uncaring items, the Unemotional items loaded more strongly on their specific, rather than the general factor. Hence, those items used to measure Unemotional traits in the ICU represent some aspects of temperament that are distinct from the more general CU construct. In line with this idea, additional analyses (see Cholesky decomposition in the Supplementary material) showed that the genetic factors underlying the Unemotional dimension are to a large degree distinct from those behind Callousness-Uncaring. This partial aetiological independence of the Unemotional subscale may explain why this dimension has not consistently demonstrated associations with the same external correlates than Callousness and Uncaring dimensions (Kimonis *et al.* 2008; Hawes *et al.* 2014).

It has also been suggested that the Unemotional dimension captures a phenotype which does not *specifically* constitute a risk for persistent antisocial behaviour and later psychopathy (Kimonis *et al.* 2013). While traditional conceptualizations of psychopathy include features related to a lack of emotion, there has been less consideration of precisely what is deemed ‘unemotional.’ For instance, while a lack of emotional *responsivity* to others' distress has been found indicative of CU features, there is less support to indicate that individuals displaying these features are devoid of emotion (e.g. frustration, anger; Blair, 2013). Therefore, it is possible that ICU items focusing on emotion are not sufficiently precise to capture atypical emotional responses related to CU features. The items that are used to assess the Unemotional dimension in the ICU quantify behaviours that are also displayed in a large array of phenotypes encompassing autism (e.g. ‘It is easy for others to tell how he/she is feeling’), depression/anhedonia (e.g. ‘He/she is very expressive and emotional’), and even anxiety/neuroticism (e.g. ‘He/she expresses his/her feelings openly’). Our research is in line with past studies, which indicate that the current Unemotional scale may not fully capture unemotionality as it relates to psychopathic presentation in youth. For example, one hallmark of psychopathic presentation is not being moved by other people's distress or joy. It may thus be of interest to formulate a new set of Unemotional items that specifically relate to unemotionality in interpersonal contexts. Whether this new set of items would relate more strongly to the Callous and Uncaring dimension and constitute a specific risk for persistent antisocial behaviour and later psychopathy (and relate more strongly to Callousness and Uncaring dimensions) could then be tested. As it stands, the current assessments of CU traits – including the ICU – may capture a construct best described as Callous-Uncaring rather than Callous-Unemotional.

### Limitations

The present study is the first genetically informative report on the ICU. This study benefited from a larger sample than those employed in all of the past CFA studies of the ICU. Yet, several limitations call for cautious interpretation of the present results. First, the ICU measure in the current study was based on parental ratings. Encouragingly, recent reports have shown the psychometric properties of the parent-report ICU to be appreciable in terms of internal consistency (e.g. Latzman *et al.* 2013) and predictive validity (White *et al.* 2009). Nonetheless, our results should be extended to multi-informant genetically informative designs. Second, several items on the Callousness-Uncaring subscale did not load strongly on any factor, which may have contributed to suboptimal model fit. While we removed two of them in our analyses (2, 10) several others (4, 7, 12, 21) did not load highly (<0.30) on any factor. Most of these items were targeted as problematic in past CFA studies (e.g. Byrd *et al.* 2013; Hawes *et al.* 2014). As these items represent virtually one quarter of the total scale, it may be beneficial to conduct further studies to systematically investigate item inclusion and possible item rewording.

### Implications

The present study highlights the usefulness of a single construct arising from the ICU, as evidenced by substantial shared genetic and environmental risk (i.e. common aetiology) across the ICU scale. The general factor is likely useful in distinguishing clinically meaningful and aetiologically homogeneous subgroups of antisocial youths (i.e. antisocial youths with high *v*. low CU traits). The present study also questions the usefulness of Unemotional traits, as currently measured, for assessing CU traits in youth and adding to the prediction of persistent antisocial behaviour and later psychopathy. These findings have implications for clinicians, as they suggest that it may be more beneficial to focus on the Callousness and Uncaring features when subgrouping youth with conduct problems

## Supplementary material

For supplementary material accompanying this paper visit http://dx.doi.org/10.1017/S0033291715001919. click here to view supplementary material
