# Supplementary material for: Genetic and environmental aetiology of the dimensions of Callous-Unemotional traits
Source: Psychol Med. 2015 Oct 12;46(2):405–14. doi: 10.1017/S0033291715001919 (PMC4682480; doi:10.1017/S0033291715001919)
Supplement: Supplementary file 1 [file S0033291715001919sup001.docx]

**SUPPLEMENTARY MATERIAL**

**Sample demographics**

13,722 families returned data for first contact on TEDS. Supplementary Table S1 (next page) presents the main demographics of this sample. Details are also provided on the study sample. Further details on the general aims, sample and methods of TEDS may be found elsewhere (Haworth, Davis, & Plomin, 2013; Olivier & Plomin, 2007; Trouton et al., 2002;).

Table S1. *Sample demographics of the Twins Early Development Study (TEDS)*.

|  | *N* families | % European descent | % Mothers with A-levels or higher | % Mother employed | % Father employed | % Female | % MZ |
| --- | --- | --- | --- | --- | --- | --- | --- |
| UK census^1^ |  | 93% | 32% | 49% | 89% | - | - |
| First contact | 13,722 | 91.7% | 35.5% | 43.1% | 91.6% | 50.1% | 33.2% |
| Study sample | 2,546 | 95.9% | 42.7% | 49.0% | 94.2% | 53.0% | 35.4% |

*Note*. ^1^Data for the 2000 UK census come from Walker et al.^5^(2001). A-levels are the national educational exam taken at 18 years of age in the UK, and refer to parental educational qualifications. MZ = monozygotic twins.

**Alternative factor structures**

Six candidate factor structures were tested. First, a one factor model (i.e., all items loading on a single factor) was tested. Second, a three correlated factors model (i.e., items of each ICU dimension, loading on a factor representing this dimension; all factors are intercorrelated) was tested. This model included the above-mentioned Callousness, Uncaring and Unemotional factors. Third, a two correlated factors model was tested. This model included a first factor termed Callousness-Uncaring, encompassing items from both subscales, and a second one termed Unemotional. Fourth, a hierarchical model with three subfactors (i.e., items of each dimension loading on a factor representing this dimension; all factors loading on a higher factor representing an overall CU dimension) was tested. Fifth, a hierarchical model with two subfactors (i.e., Callousness-Uncaring and Unemotional) was tested. Sixth, a general-specific model, including the Callousness, Uncaring and Unemotional as specific factors, was tested. A seventh model was tested according to a general-specific structure, this time including only two specific factors: Callousness-Uncaring and Unemotional.

Factorial structures including two, rather than three, specific factors were tested as previous research suggested that: 1) the Callousness and Uncaring dimensions behave similarly in terms of external correlates and factor loadings (Byrd et al., 2013; Kimonis et al., 2008; Roose et al., 2010), and that; 2) several ICU items allocated to Callousness and Uncaring in past studies are very similar in content (e.g., item 5 from the Callousness subscale “He/she feels bad or guilty when he/she does something wrong, reversed” vs. item 18 from the Uncaring subscale “He/she does not feel remorseful when he/she does something wrong.”; e.g., Essau et al., 2006).

**Model fit indices**

Model fit was assessed using the following indices: chi square goodness of fit statistic (*χ*^2^); Akaike’s Information Criterion (AIC); Scaled Comparative Fit Index (CFI); Scaled Root Mean Square Error of Approximation (RMSEA); Standardized Root Mean Square Residual (SRMR). The *χ*^2^ is a function of the sample size and the difference between the observed and model covariance matrices. Akaike information criterion (AIC) is an index for model comparison, in which smaller values indicate better fit (Akaike, 1987). Different values of CFI have been suggested to indicate adequate fit (e.g., ≥ .90; Segars & Grover, 1993; ≥ .95; Hu & Bentler, 1999). The RMSEA provides a fit index unaffected by the size of the model. A RMSEA value of .06 is considered acceptable (Hu & Bentler, 1999). The SRMR is an absolute fit indicator and a value smaller than .08 is acceptable (Hu & Bentler, 1999). On the basis of CFA analyses, we selected the candidate factorial structure for genetic modelling.

**Multivariate genetic model**

As intraclass correlations for the total ICU score and the Callousness-Uncaring score were indicative of substantial heritability (see Results), a traditional ACE structure was tested for both the general and Callousness-Uncaring factors. Intraclass correlations on the Unemotional score indicated that an ADE structure – the D component standing for non-additive genetic variance – would be best suited for the Unemotional factor (i.e., *r_dz_* < ½ *r_mz_*). However, twin zygosity differences on the Unemotional scores were detected in preliminary analyses (twin 1: V_MZ_ = 7.51; V_DZ_ = 9.27; twin 2: V_MZ_ = 7.56; V_DZ_ = 8.99). This suggests that sibling interaction effects could partly account for the discrepancy between MZ and DZ intraclass correlations. We therefore: 1) tested a full multivariate genetic model including an ADE structure for the Unemotional factor, as well as sibling interaction effects on this factor; 2) tested a full multivariate genetic model including an AE structure for the Unemotional factor (without a D component), as well as sibling interaction effects on this factor, and; 3) compared the fit of models 1) and 2) in order to test the statistical significance of the Unemotional factor’s D component. Comparison of models 1) and 2) indicated that, in a full model including sibling interaction effects on the Unemotional factor, the D component was not statistically significant. Therefore, an AE structure was tested in the Unemotional factor (see Figure 1). Similar steps were taken for the Cholesky decomposition, and this endeavour also revealed than the D component was not statistically significant for the Unemotional subscale score.

**Cholesky decomposition**

A Cholesky decomposition was also fitted on the Callousness-Uncaring and Unemotional scores of the ICU. The aim of this complementary analysis was to verify to what extent the genetic contributions on the Unemotional score were unique to this dimension or explained by the Callousness-Uncaring dimension. The traditional ACE structure was tested for the Callousness-Uncaring scores. Intraclass correlations warranted an ADE structure for the Unemotional scores. However, as stated earlier, the D component (i.e., non-additive genetic variance) was largely driven by sibling interaction effects on the Unemotional measure (for more details, see the section Multivariate genetic model in the Other Supplementary Material). Consequently, an AE structure was tested in the context of this Cholesky decomposition. Sibling interaction effects are included for the Unemotional score.

As can be seen in Supplementary Table S2, genetic factors explained 78% of the total variance in the Unemotional score. These genetic contributions were largely specific to the Unemotional score (78%), rather than explained by the Callousness-Uncaring score (22%). A similar pattern was observed for nonshared environmental factors. Shared environmental factors were not statistically significant for the Callousness-Uncaring score.

Table S2. *Cholesky decomposition of heritability, shared environment, and nonshared environment for the ICU dimensions.*

|  | A1 [CI_lower_-CI_upper_] | | A2 [CI_lower_-CI_upper_] |  | Total *h*^2^ [CI_lower_-CI_upper_] |
| --- | --- | --- | --- | --- | --- |
| CL-UC | **.77** [**.68-.84**] |  | |  | .77 [.68-.84] |
| UE | **.17** [**.14-.19**] | **.61** [**.57-.65**] | |  | .78 [.74-.82] |
|  | C1 [CI_lower_-CI_upper_] | C2 [CI_lower_-CI_upper_] | |  | Total *c*^2^ [CI_lower_-CI_upper_] |
| CL-UC | .05 [.00-.12] |  | |  | .05 [.00-.12] |
|  | E1 [CI_lower_-CI_upper_] | E2 [CI_lower_-CI_upper_] | |  | Total *e*^2^ [CI_lower_-CI_upper_] |
| CL-UC | **.18** [**.14-.20**] |  | |  | .18 [.14-.20] |
| UE | **.01 [.01-.02]** | **.21 [.17-.25]** | |  | .22 [.18-.26] |

*Note*. CL-UC = Callousness-Uncaring; UE = Unemotional; [CI_lower_-CI_upper_] = 95% confidence intervals. The values presented in the table are standardized components of variance. Significant paths are in bold. Fit indices for the model: χ^2^ = 73.34, *df* = 18, *p* < .0001; AIC = 100185.466; CFI = .98; SRMR = 0.05; RMSEA [95% CI_lower_-CI_upper_] = .04 [.03-.04]. Genetic correlation = .46, *p* < .001; Bivariate heritability = .357, *p* < .001;; Nonshared environmental correlation = .25, *p* < .001; Bivariate Nonshared environmental contributions = .05, *p* < .001. An ACE structure was tested on the CL-UC factor, while an AE structure was tested on the UE factor. Sibling interaction effects are included for the Unemotional score.
